# 818 Pediatric Fire-Related Mortality: A Retrospective Review of National Fatality Review Case Reporting System Data

**DOI:** 10.1093/jbcr/iraf019.349

**Published:** 2025-04-01

**Authors:** Francis Pleban, Howard Needelman

**Affiliations:** Tennessee State University; Nebraska Medical Center

## Abstract

**Introduction:**

This study explored the National Fatality Review-Case Reporting System (NFR-CRS) as a data source to describe pediatric fire-related residential (FRR) deaths among children aged 0-17 years during the period 2004 to 2020.

**Methods:**

Fire-related residential deaths were selected from the NFR-CRS if the child’s primary cause of death was a fire that occurred at the child’s home, a relative’s home, or a friend’s home. Frequencies and percentages were calculated to describe child demographics, primary caregiver characteristics, incident circumstances, and family environmental characteristics.

**Results:**

In this study, 2,631 children who died in residential fires were identified. Unadjusted results revealed a predominant male (55.0%) and White (57.3%), primarily aged 1-4 (42.6%). Over three-quarters (77.7%) of the children were under 10 years old, with the highest proportion falling within the 1-4 age group. Most incidents occurred within the child’s home (86.4%). The majority of affected residences were single-family houses (51.4%), followed by trailer/mobile homes (18.7%) and multi-unit dwellings (17.3%). Pediatric fatalities were often concentrated between 11 pm and 4 am (35.2%) and were predominantly accidental (83.9%). Notably, 51.8% of fire-related deaths involved more than one child aged 0-18 years. The child’s primary caregiver at the time of death was typically the biological parent (75.3%), aged 25-34 (32.9%), female (81.0%), and English-speaking (75.7%).

**Conclusions:**

This study explored the NFR-CRS as a data source to describe pediatric FRR deaths among children aged 0-17 years during the period 2004 to 2020. Certain limitations exist regarding the use of NFR- CRS data. The NFR-CRS only includes infant and child deaths reviewed by fatality review teams. Data quality presents another concern, encompassing issues such as inconsistent data, delayed data entry, and overuse of the “Other” specified field, which can impact the overall generalizability of the data.

**Applicability of Research to Practice:**

Compiling and disseminating Child Death Review (CDR) data through comprehensive reports may represent one strategy for informing policy makers, agency personnel, and the general public about critical risk factors and prevention opportunities. The National Fatality Review-Case Reporting System (NFR-CRS), established in 2005, offers a standardized framework for documenting and analyzing cases. CDR practitioners utilize this system to access data, synthesize findings, and generate uniform reports. The examination of child fatalities during case reviews often catalyzes localized and statewide initiatives aimed at preventing similar deaths. Systematic data collection and ongoing reporting play pivotal roles in this process, while comparing review outcomes with infant and child mortality data from official records enhances the robustness of these findings.

**Funding for the Study:**

N/A

